# Response of Pine Rhizosphere Microbiota to Foliar Treatment with Resistance-Inducing Bacteria against Pine Wilt Disease

**DOI:** 10.3390/microorganisms9040688

**Published:** 2021-03-26

**Authors:** Gil Han, Mohamed Mannaa, Namgyu Kim, Hee Won Jeon, Hyejung Jung, Hyun-Hee Lee, Junheon Kim, Jungwook Park, Ae Ran Park, Jin-Cheol Kim, Young-Su Seo

**Affiliations:** 1 Department of Integrated Biological Science, Pusan National University, Busan 46241, Korea; croone@pusan.ac.kr (G.H.); mannaa_mohamed@yahoo.com (M.M.); titanic622@pusan.ac.kr (N.K.); jhj4059@pusan.ac.kr (H.J.); ehyuna92@pusan.ac.kr (H.-H.L.); jjuwoogi@pusan.ac.kr (J.P.); 2 Department of Plant Pathology, Cairo University, Giza 12613, Egypt; 3 Division of Applied Bioscience and Biotechnology, Chonnam National University, Gwangju 61186, Korea; jeon-hw@naver.com (H.W.J.); arpark9@naver.com (A.R.P.); 4 Forest Insect Pests and Diseases Division, National Institute of Forest Science, Seoul 02455, Korea; junheonkim@korea.kr; 5 Environmental Microbiology Research Team, Nakdonggang National Institute of Biological Resources (NNIBR), Sangju 37242, Korea

**Keywords:** induced resistance, rhizosphere microbiota, pine wilt disease, pine wood nematode, biocontrol

## Abstract

In this study, two bacterial strains, IRP7 and IRP8, were selected to induce resistance against pine wilt disease (PWD). Foliar application with these strains to nematode-inoculated pine seedlings significantly reduced PWD severity. The effect of nematode inoculation and bacterial treatment on the rhizosphere bacterial community was investigated. The results indicated that the rhizosphere of nematode-inoculated seedlings contained a lower relative abundance of beneficial microbes such as *Paraburkholderia*, *Bradyrhizobium*, *Rhizobacter*, *Lysobacter*, and *Caballeronia.* Bacterial treatment resulted in significant changes in the microbes that were represented in relatively low relative abundance. Treatment with IRP7 resulted in an increase in the relative abundance of *Nitrospirillum*, *Bacillus*, and *Luteibacter*, which might be useful for protection against infection. Treatment with IRP8 resulted in an increase in the relative abundance of obligate bacterial predators of the *Bdellovibrio* genus that were previously shown to control several bacterial phytopathogens and may have a role in the management of nematode-carried bacteria. The selected bacteria were identified as *Pseudomonas koreensis* IRP7 and *Lysobacter enzymogenes* IRP8 and are suggested as a potential treatment for induced resistance against PWD. To our knowledge, this is the first report on the effect of foliar treatment with resistance-inducing bacteria on the rhizosphere microbiota.

## 1. Introduction

Pine wilt disease (PWD) is a major threat to pine forests worldwide, causing huge economic and environmental losses due to widespread infections of pine trees [1]. The disease is mainly caused by the pine wood nematode (PWN) *Bursaphelenchus xylophilus*, first reported in Japan in 1971, although disease symptoms were observed as early as 1905. Moreover, PWD has rapidly spread to several Asian and European countries and is currently considered a quarantine pest in many countries owing to the remarkable level of destruction of pine trees and high mortality rates [2,3,4].

In addition to PWN, there is evidence of the important roles of the pine sawyer longhorn *Monochamus* beetle vector, microbes carried by nematodes, and the associated ophiostomatoid blue stain fungi in disease development, which present PWD as a model for plant pathobiome studies [5]. Bacteria carried by nematodes, such as *Bacillus* and *Burkholeria* spp., produce phytotoxins involved in the development of disease symptoms and browning of tissues [6,7,8,9]. It was also reported that inoculation of pine trees with microbe-free axenic nematodes could not induce PWD symptoms [10]. In contrast, beneficial plant-associated microbes play key roles in supporting plant health and protecting trees against infection through many approaches including induction of resistance or direct nematocidal activity [11].

To restrict the spread of the disease, governments in affected countries are urged to follow strict quarantine regulations and measures, including detection; monitoring; conducting surveys; establishing quarantine and clear-cut zones around affected regions; treatment, fumigation, and eradication of affected trees; and restricting wood transportation from affected areas [12]. Several methods, including physical chipping and burning, targeting the insect vector with traps, and chemical and biological methods, have been studied and tested for the management and control of PWD. Chemical methods include the injection of trees with nematicides such as abamectin and emamectin benzoate, which are useful for limiting the spread of PWD [13,14].

Owing to rising concerns regarding the hazardous effects of chemical treatment on human health and the environment, as well as the impracticability of large-scale applications, environmentally friendly solutions are needed [15]. Biological control has been proposed against PWN, the insect vector, and nematode-carried bacteria [12]. The nematode endoparasitic fungus, *Esteya vermicola*, was shown to be effective for in vitro biocontrol of PWN and has also been proven effective for significantly reducing and delaying pine tree infection for more than 5 years [16,17]. The endoparasitic fungus was reported to attract PWN to fungal spores in vitro and in pine seedlings by producing specific chemical lure substances [16,18]. In addition, 1-aminocyclopropane-1-carboxylate deaminase-producing *Pseudomonas putida* was suggested as a potential biocontrol agent against PWD because the treatment significantly reduced typical symptoms [19]. Natural enemies of the sawyer beetle, such as *Scleroderma guani*, were used after mass rearing in the laboratory to control the insect vector, particularly after clear-cutting of infected dead trees, and resulted in a significant reduction in PWD incidence [20].

Induction of resistance represents another promising control strategy that has been confirmed to be effective in several previous studies. Early studies in Japan have reported that pre-inoculation with an avirulent strain of the nematode induced systemic resistance in pine trees against subsequent inoculation with virulent strains with significantly higher survival rates compared with those in the control, particularly when multiple pre-inoculations with the avirulent strains were performed [21]. Three bacterial strains, *P. putida* 16YSM-E48, *Curtobacterium pusillum* 16YSM-P180, and *Stenotrophomonas rhizophila* 16YSM-P39, were previously isolated and selected for their ability to induce systemic resistance in pine calli and seedlings [22].

To apply induced resistance in the management of PWD, the effect of treatment on different environmental aspects should be investigated. Mannaa et al. [23], reported the induction of systemic resistance in pine seedlings using resistance-inducing chemical elicitors methyl salicylic acid (MeSA) and acibenzolar-s-methyl (ASM), which resulted in significant shifts in several ecologically important microbial taxa within the pine rhizosphere microbiota. The objective of the current study was to evaluate the effect of foliar treatment with resistance-inducing bacterial species on PWD severity and to use high-throughput next-generation sequencing-based analysis to evaluate their effect on the microbial composition of the pine seedling rhizosphere. The selected bacterial strains in this study were identified at the species level and may serve as a potential environmentally friendly option for the management of PWD.

## 2. Materials and Methods

### 2.1. Preparation of Bacterial Treatment and Nematode Inoculum

Twelve bacterial strains were isolated from pine forests in South Korea and were selected for their ability to induce resistance-related genes in vitro (Figure S1). The bacterial strains IRP7 and IRP8 were selected based on the reduction of PWD severity in preliminary experiments. These strains were stored at −80 °C and cultured in tryptic soy agar (TSA) at 28 °C for 48 h, and single colonies were transferred to tryptic soy broth (TSB) and incubated in a shaking incubator (150 rpm) at 28 °C for 48 h. Bacterial culture concentrations were adjusted to OD_600_ = 0.8 for treatment.

The highly virulent causal agent of PWD *B. xylophilus* was obtained from the National Institute of Forest Science (Seoul, South Korea). The inoculum was prepared by inoculating *Botrytis cinerea* nematode cultured on potato dextrose agar for 1 week at 25 °C. After incubation, the PWN was separated from the fungal culture using the Baermann funnel method [24]. The PWN suspension was adjusted to a concentration of 20,000 nematodes/mL in sterile distilled water (SDW). Pine seedlings (*Pinus densiflora*) used in this study were purchased from Daelim Seedling Farm, Okcheon, South Korea.

### 2.2. Pine Seedling Assay

Three-year-old *P. densiflora* seedlings were used for the pine seedling assay to evaluate the effect of treatment with IRP7 and IRP8 bacterial strains against PWD. Briefly, pine seedlings grown under greenhouse conditions were foliar sprayed twice at 1-week-intervals with 5 mL of the bacterial suspension prepared as explained above. One week after treatment, seedlings were inoculated with PWN as described in a previous study [25]. The PWN water suspension (2000 nematode/100 mL) was pipetted into an absorbent cotton inserted into a small slit made in pine seedlings with a surface-sterilized knife. The inoculated cotton was then wrapped to prevent drying using Parafilm M (Heathrow Scientific, Vernon Hills, IL, USA). Control seedlings were treated with SDW, and five replicates were maintained for each treatment. At 30 days after inoculation, disease severity was evaluated using a 5-degree scale as follows: 0 = healthy seedlings showing no wilting or needle-browning symptoms, 1 = <20% needle browning, 2 = 20–39% needle browning, 3 = 40–59% needle browning, 4 = 60–79% needle browning and terminal shoots bending, and 5 = 80–100% needle browning and wilting of whole seedling [22].

### 2.3. Pine Rhizosphere and Soil Metagenomic DNA Extraction, Sequencing, and Bioinformatics Analysis

Samples were collected from the roots and closely attached soil around the roots of each seedling. From each replicate, a composite sample from different sides and depths of each seedling was mixed. The collected samples (~10 g) were dissolved in 20 mL SDW and vigorously vortexed before being centrifuged at 10,000× *g* for 15 min to remove excess water. Metagenomic DNA extraction was performed with the PowerSoil^®^ DNA Isolation Kit (MO BIO Laboratories, Carlsbad, CA, USA) using 250 mg of the collected pellets, according to the manufacturer’s instructions. The quality and concentration of the isolated metagenomic DNA were evaluated by gel electrophoresis and a NanoDrop2000 spectrophotometer (Thermo Fisher Scientific, Wilmington, NC, USA). Qualified metagenomic bacterial DNA samples were stored until use in Tris-EDTA buffer solution at 20 °C.

The pine rhizosphere and soil microbial community meta-barcoding analysis were based on sequencing of the V3 and V4 variables of the 16S rRNA region. Polymerase chain reaction (PCR) amplification, sequence analysis, and library preparation were performed according to the Herculase II fusion DNA polymerase Nextera XT Index Kit V2 protocol of the Illumina^®^ MiSeq^®^ platform at Macrogen (Seoul, South Korea) using the following primer pair:

(F) 5′-TCGTCGGCAGCGTCAGATGTGTATAAGAGACAGCCTACGGGNGGCWGCAG-3′;

(R) 5′-GTCTCGTGGGCTCGGAGATGTGTATAAGAGACAGGACTACHVGGGTATCTAATCC-3′.

The resulting paired-end reads were merged using the fast length adjustment of short reads (FLASH; http://ccb.jhu.edu/software/FLASH, accessed on 15 January 2021) [26]. The Illumina adaptors and short- and low-quality reads were removed from the raw sequences using Scythe (v0.994) (https://github.com/vsbuffalo/scythe, accessed on 15 January 2021) and Sickle software (https://github.com/najoshi/sickle, accessed on 15 January 2021). The CD-HIT-OTU-MiSeq and UCLUST algorithms were used for clustering and annotation of the preprocessed sequences, which were then arranged into the respective operational taxonomic units (OTUs) using the Greengenes database at a cut-off value of 97% [27,28,29]. Microbiome analyses from sequence annotation and diversity statistics for the taxonomic assignments of the obtained OTUs were performed using the Quantitative Insights into Microbial Ecology version 2 (QIIME2) pipeline [30]. The obtained sequences were deposited as a sequence read archive in the National Center for Biotechnology Information database under BioProject ID PRJNA689106.

### 2.4. Taxonomic Identification of Resistance-Inducing Bacterial Strains IRP7 and IRP8

The bacterial strains IRP7 and IRP8 were identified at the species level based on 16S rRNA sequence analysis. Bacterial genomic DNA was extracted from cultures of both strains that were incubated overnight in TSB at 28 °C in a shaking incubator (150 rpm). The Wizard Genomic DNA Purification Kit (Promega, Madison, Wisconsin, USA) was used for DNA extraction following the manufacturer’s instructions. Nearly complete 16S rRNA sequence was amplified by PCR using the universal primer pairs fD1 (5′-AGAGTTTGATCCTGGCTCAG-3′) and rP2 (5′-ACGGCTACCTTGTTACGACTT-3′) [31]. The obtained 16S rRNA sequences were analyzed using the basic local alignment search tool, compared with 16S rRNA sequences from related type strains, and a phylogenetic tree based on maximum likelihood was constructed using Mega X software [32].

### 2.5. Statistical Analysis

Disease severity from the seedling assay data was evaluated using analysis of variance through the general linear model procedures conducted using the Statistical Analysis System (SAS Institute, Cary, NC, USA), and means were separated using the least significant difference test at *p* < 0.05. Pine rhizosphere and soil microbiome diversity and rarefaction were estimated using QIIME2 scripts, R (version 3.1.3), and the PAleontological STatistics software package (PAST) version 3.23 [33]. Principal coordinate analysis (PCoA) was performed based on weighted and unweighted UniFrac distances.

## 3. Results

### 3.1. Foliar Treatment with Selected Bacterial Strains Reduced Pine Wilt Disease (PWD) Severity in Inoculated Seedlings

**Figure 1:**
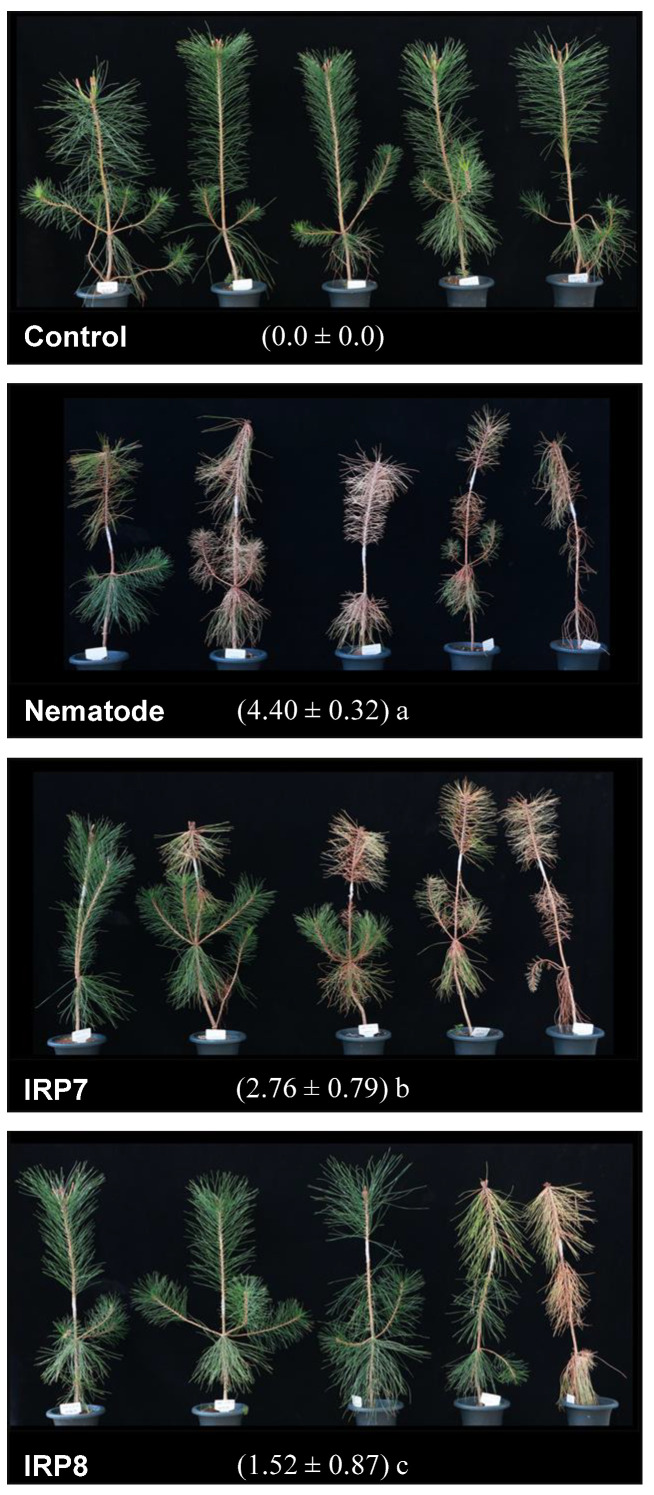
Effect of resistance-inducing bacterial treatment on pine wilt disease. Control, Untreated and uninoculated pine seedlings; Neamtode, pine seedlings inoculated with nematode, *Bursaphelenchus xylophilus*; IRP7, nematode-inoculated pine seedlings treated with *Pseudomonas koreensis* IRP7; IRP8, nematode-inoculated pine seedlings treated with *Lysobacter enzymogenes* IRP8. Disease severity at 30 days post-inoculation is presented under each photograph as the mean ± standard deviation of five replicates; different lowercase letters following the disease severity indicate statistical significance at *p* < 0.05 according to the least significant difference test.

At 30-days post inoculation, IRP7- and IRP8-treated seedlings showed significantly reduced disease severity compared with untreated nematode-inoculated seedlings, which showed browning and wilting of the needles that were mostly bent (Figure 1). Seedlings treated with IRP8 showed the best control efficacy compared with other treatments, as shown in the seedling photographs and as indicated by the evaluated disease severity (Figure 1).

### 3.2. Sequencing Results and Sequence Analysis

High-throughput sequencing of the 24 pine rhizosphere metagenomic samples resulted in a total of 2,408,243 reads, with an average of 100,343 reads per sample. The obtained raw sequences, total base count, read count, GC%, Q20%, and Q30% for the used samples are shown in Table S1. A total of 567,708 reads, with an average of 23,654 reads per sample (minimum of 17,070.0 and maximum 28,468.0) were obtained following the screening of low-quality short reads using CD-HIT-OTU.

**Figure 2:**
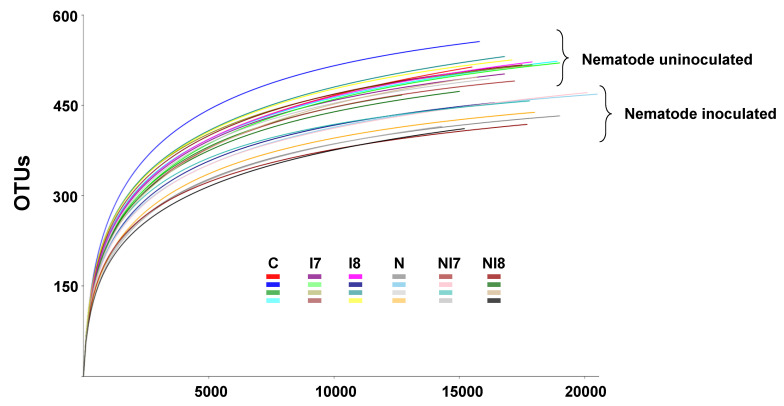
Operational taxonomic unit (OTU) rarefaction curves of the 16S rRNA sequence reads obtained revealing satisfactory sequencing depth to represent the pine rhizosphere and soil microbiota. C, negative control; I7, samples treated with *Pseudomonas koreensis* IRP7; I8, samples treated with *Lysobacter enzymogenes* IRP8; N, nematode-inoculated; NI7, nematode-inoculated samples treated with *P. koreensis* IRP7; NI8, nematode-inoculated samples treated with *L. enzymogenes* IRP8.

Sequencing depth was confirmed to be sufficient to represent the major components of the bacterial diversity in the rhizosphere communities by plotting rarefaction curves of the OTU number to the obtained sequence reads, which indicated near-leveling of the curves at around 6000 reads (Figure 2).

On comparing the alpha diversity indices among the tested samples, no significant differences were observed, except that the inverse Simpson index for the pine seedlings treated with IRP8 strain was lower than that of the other groups and the Shannon index for the pine seedlings treated with IRP7 and IRP8 strains after inoculation with the nematode were lower than those of the other groups. Moreover, when the seedlings inoculated with the nematode were compared with the uninoculated seedlings regardless of the bacterial treatment, the estimated OTUs, Chao1, and Shannon diversity indices were significantly lower in the nematode-inoculated seedlings (Table 1).

**Figure 3:**
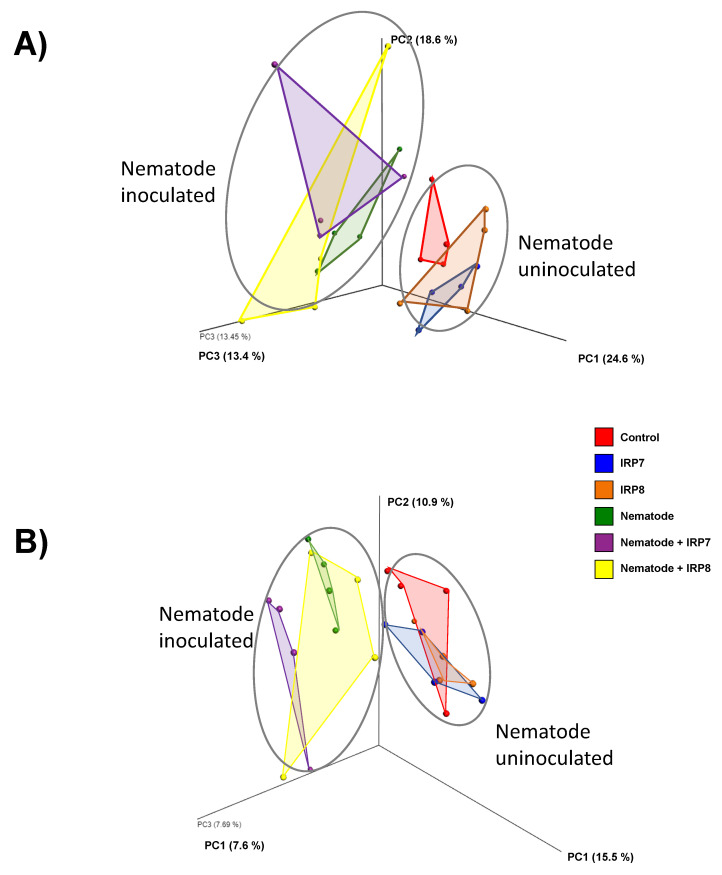
Three-dimensional graphs showing the principal coordinate analysis (PCoA) of the (**A**) weighted and (**B**) unweighted UniFrac distances from the pine rhizosphere microbiota. C, negative control; IRP7, samples treated with *Pseudomonas koreensis* IRP7; IRP8, samples treated with *Lysobacter enzymogenes* IRP8; N, nematode-inoculated; Nematode + IRP7, nematode-inoculated samples treated with *P. koreensis* IRP7; Nematode + IRP8, nematode-inoculated samples treated with *L. enzymogenes* IRP8. A clear distinction was observed between the nematode-inoculated and uninoculated samples that were separated into distinct clusters.

Regarding the beta diversity among the different groups of pine seedling rhizosphere microbiota, a clear distinction was observed between the nematode-inoculated seedlings and uninoculated groups based on the PCoA of the weighted and unweighted UniFrac distances; however, bacterial treatment showed less effect compared with that with nematode inoculation (Figure 3A,B).

### 3.3. Pine Rhizosphere Bacterial Composition Structure Affected by Nematode Inoculation and Bacterial Treatment

**Figure 4:**
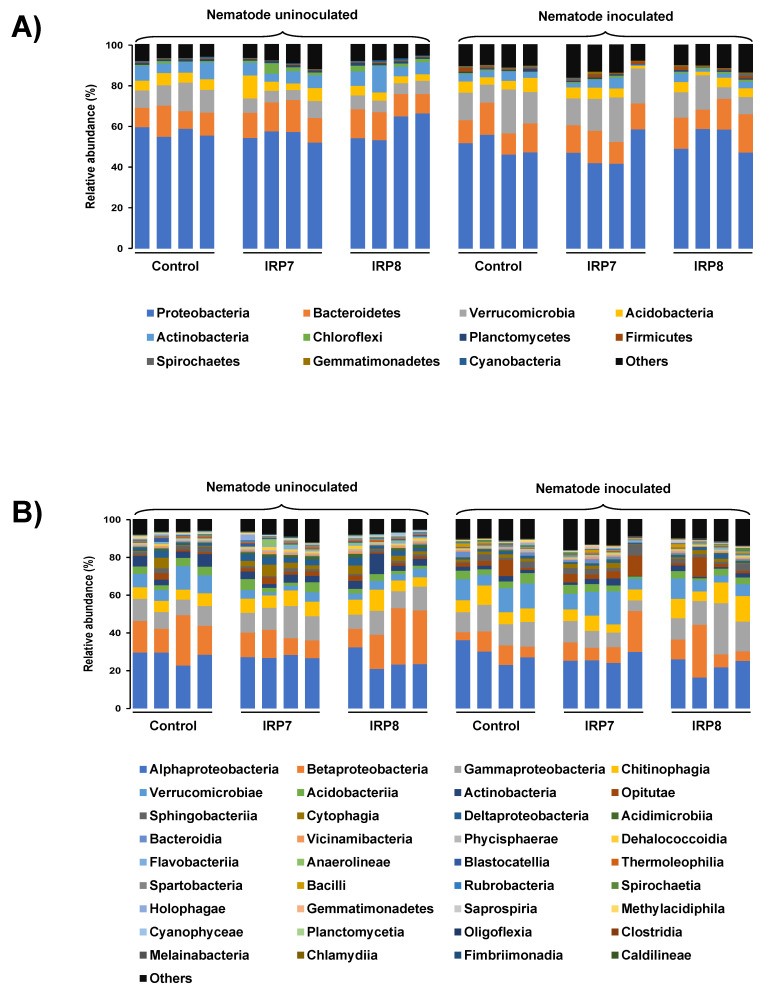
Stacked bar graph for the relative abundance of bacterial 16S rRNA at (**A**) the phylum and (**B**) class levels using rhizosphere samples of the nematode (*Bursaphelenchus xylophilus*)-inoculated and -uninoculated pine seedlings treated with *Pseudomonas koreensis* IRP7 or *Lysobacter enzymogenes* IRP8.

As shown in Figure 4, the bacterial community at the phylum and class levels of the pine seedling rhizosphere was dominated mainly by the Alpha-, Beta-, and Gamma-proteobacteria, followed by Bacteroidetes (mainly Chitinophagia and Sphingobacteria) and Verrucomicrobia (mainly Verrucomicrobiae and Opitutae), representing an average relative abundance of 77%.

At the phylum level, when nematode-inoculated pine seedlings were compared with the uninoculated group, the relative abundances of Proteobacteria, Actinobacteria, and Cyanobacteria were significantly lower in the nematode-inoculated group than in the uninoculated group, whereas the Spirochaetes and Gemmatimonadetes were significantly higher in the nematode-inoculated group than in the uninoculated group. When bacterial-treated pine seedlings were compared with the untreated nematode-inoculated group, no significant changes were observed, except for the increase in the relative abundance of the Chloroflexi phylum in the IRP8-treated group.

At the class level, the relative abundance of Betaproteobacteria, Thermoleophilia, Planctomycetia, and Cyanophyceae was significantly higher in nematode-inoculated pine seedlings than in the uninoculated group, whereas the relative abundances of Spirochaetia and Gemmatimonadetes were significantly lower. The relative abundance of Gammaproteobacteria and Planctomycetia was significantly lower in the nematode-inoculated pine seedlings treated with IRP7 than in the untreated group. The relative abundance of Anaerolineae, Oligoflexia, and Fibrobacteria was significantly higher in nematode-inoculated pine seedlings treated with IRP8 than in the untreated group, whereas the relative abundance of Planctomycetia was significantly lower.

**Figure 5:**
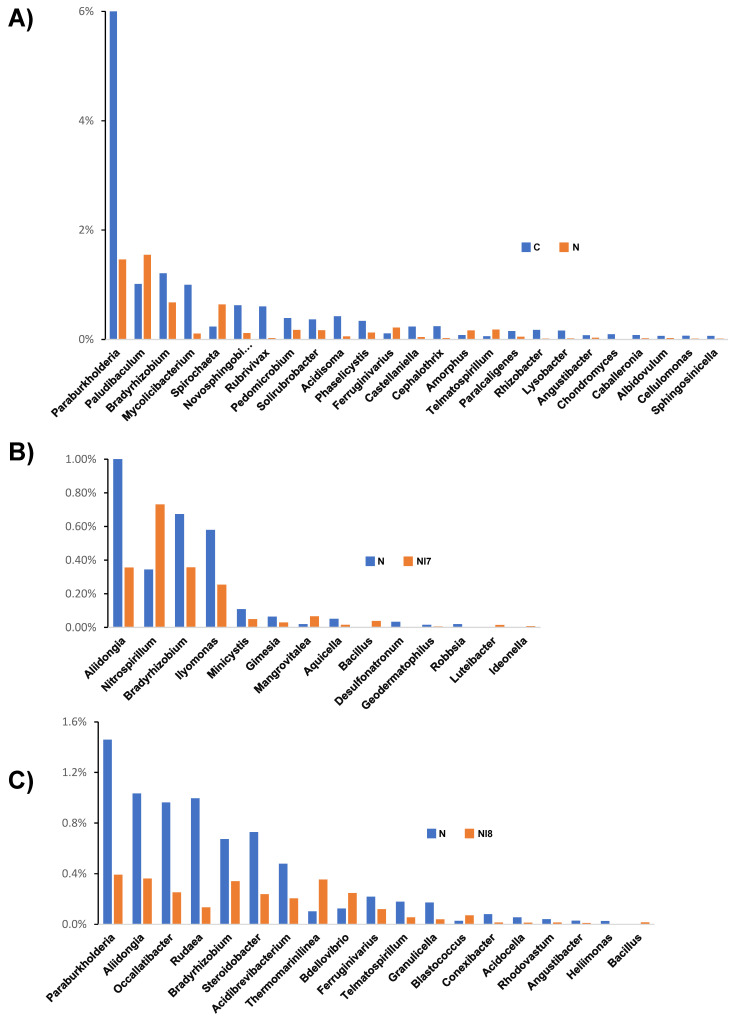
Bar graphs showing the relative abundance of rhizosphere bacterial genera from pine seedlings. Bacterial genera existed at significantly different (*p* < 0.05) relative abundance levels between (**A**) nematode-inoculated and uninoculated control pine seedlings, (**B**) nematode-inoculated control seedlings and nematode-inoculated and IRP7-treated seedlings, and (**C**) nematode-inoculated control seedlings and nematode-inoculated and IRP8-treated seedlings. The bars represent the mean values of four replicates.

The relative abundance of bacterial genera existing at significantly different levels between nematode inoculated pine seedlings and uninoculated control is shown in Figure 5A, whereas differences between IRP7- and IRP8-treated nematode inoculated pine seedlings and untreated nematode-inoculated control are shown in Figure 5B,C. The relative abundance of a greater number of bacterial genera was affected by nematode inoculation than by IRP7 or IRP8 bacterial treatment.

The relative abundance of *Paraburkholderia*, *Bradyrhizobium*, *Mycolicibacterium*, *Novosphingobium*, *Rubrivivax*, *Pedomicrobium*, *Solirubrobacter*, *Acidisoma*, *Phaselicystis*, *Castellaniella*, *Cephalothrix*, *Paralcaligenes*, *Rhizobacter*, *Lysobacter*, *Angustibacter*, *Chondromyces*, *Caballeronia*, *Albidovulum*, *Cellulomonas*, and *Sphingosinicella* were significantly lower in nematode-inoculated pine seedlings than in the uninoculated control, whereas the relative abundance of *Paludibaculum, Spirochaeta*, *Ferruginivarius*, *Amorphus*, and *Telmatospirillum* was significantly higher (Figure 5A). In the IRP7-treated nematode-inoculated pine seedlings, the relative abundance of *Nitrospirillum*, *Mangrovitalea*, *Bacillus*, *Luteibacter*, and *Ideonella* was significantly higher than that in untreated seedlings, whereas the relative abundance of *Aliidongia*, *Bradyrhizobium*, *Ilyomonas*, *Minicystis*, *Gimesia*, *Aquicella*, *Desulfonatronum*, *Geodermatophilus*, and *Robbsia* was significantly lower (Figure 5B). In the IRP8-treated nematode-inoculated pine seedlings, the relative abundance of *Thermomarinilinea*, *Bdellovibrio*, *Blastococcus*, and *Bacillus* was significantly higher than that in untreated seedlings, whereas the relative abundance of *Paraburkholderia*, *Aliidongia*, *Occallatibacter*, *Rudaea*, *Bradyrhizobium*, *Steroidobacter*, *Acidibrevibacterium*, *Ferruginivarius*, *Telmatospirillum*, *Granulicella*, *Conexibacter*, *Acidocella*, *Rhodovastum*, *Angustibacter*, and *Heliimonas* was significantly lower (Figure 5C).

**Figure 6:**
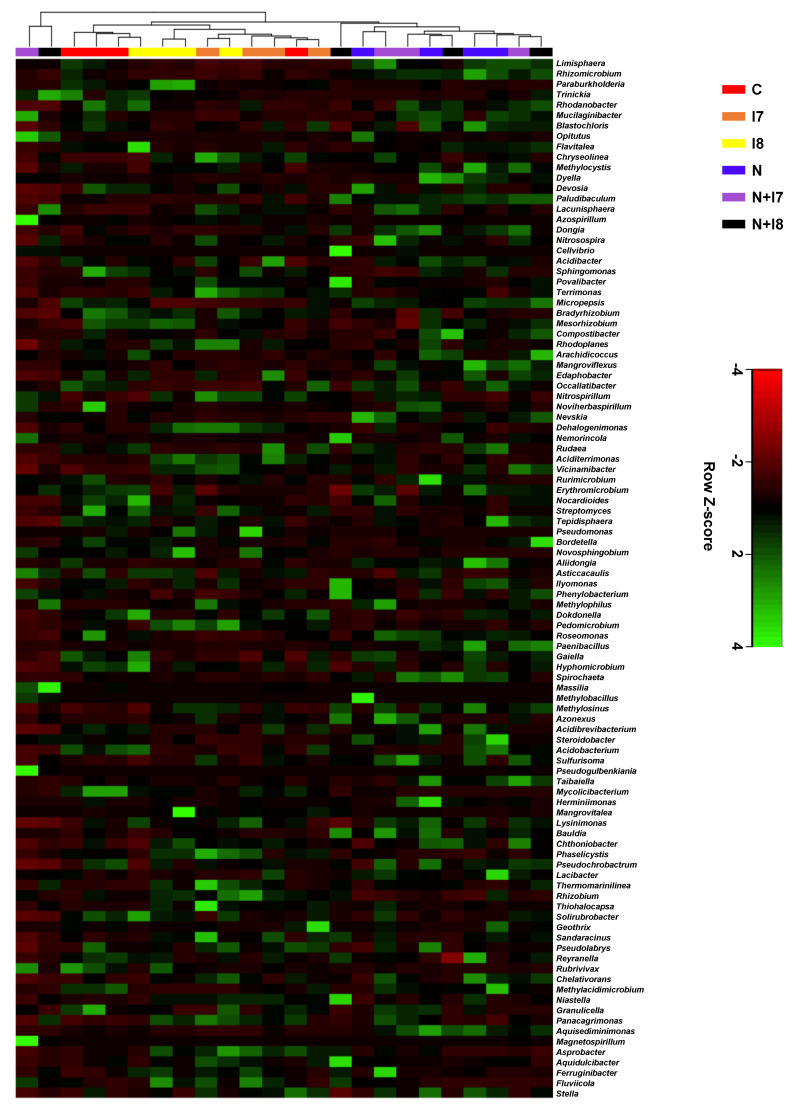
Heatmap of the complete linkage hierarchical clustering based on the Manhattan distance measurements of the 100 most abundant bacterial genera in the rhizosphere of pine seedlings. C, negative control; I7, samples treated with *Pseudomonas koreensis* IRP7; I8, samples treated with *Lysobacter enzymogenes* IRP8; N, nematode-inoculated; N+I7, nematode-inoculated samples treated with *P. koreensis* IRP7; N+I8, nematode-inoculated samples treated with *L. enzymogenes* IRP8. The standardized Z-Score represents the relative abundance of bacterial genera in each row.

A complete linkage hierarchical clustering heatmap, based on the Manhattan distance measurements, was created for the 100 most abundant bacterial genera in the pine seedling rhizosphere. The nematode-inoculated seedlings were grouped into separate clusters from the uninoculated seedlings, indicating observed differences in the microbial population regardless of treatment with IRP7 and IRP8 strains, which had relatively little effect on the microbial structure (Figure 6).

**Figure 7:**
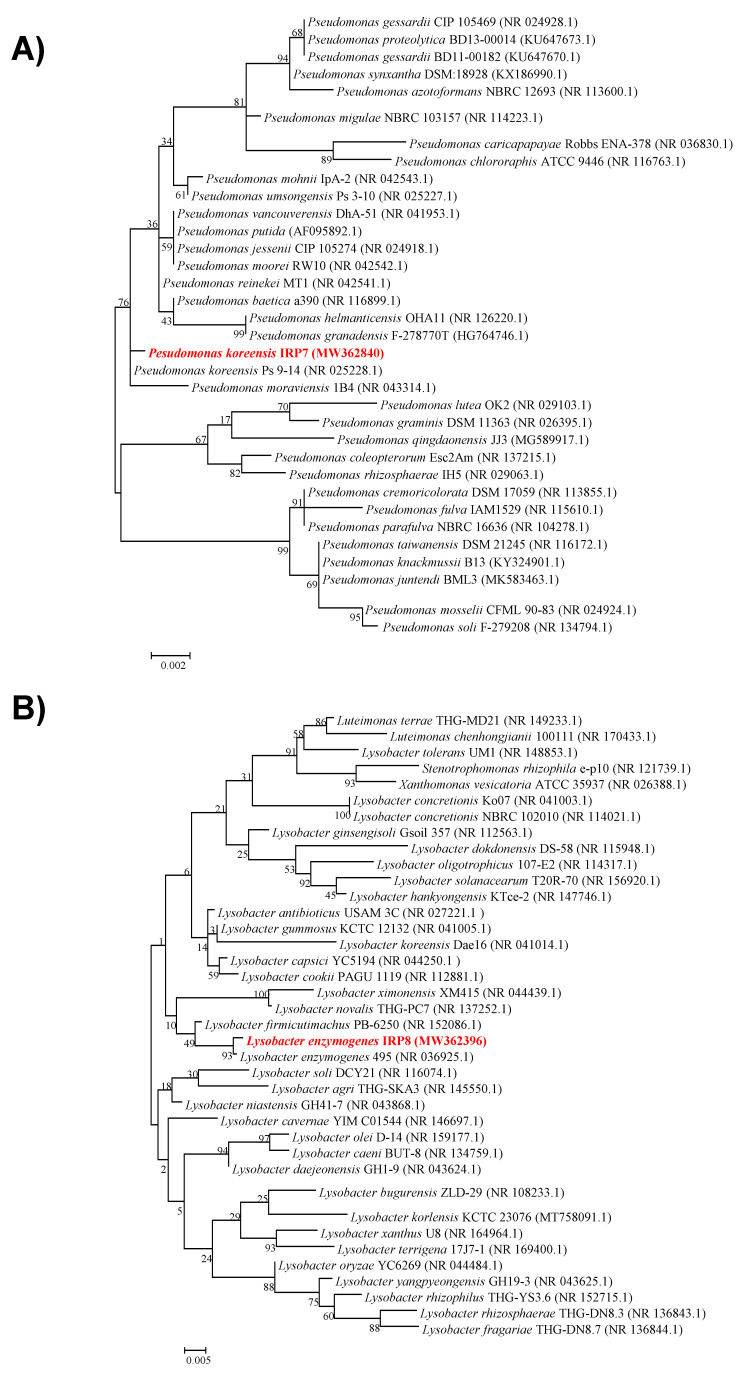
Maximum-likelihood phylogenetic tree based on the 16s rRNA sequences for taxonomic position and relationships among type strains of related species for (**A**) *Pseudomonas koreensis* IRP7 and (**B**) *Lysobacter enzymogenes* IRP8. Bootstrap values of 1000 replicates are shown on the branching points. Bacterial species names are followed by strain names and accession numbers of the sequences in parentheses.

The selected strains used in this study were identified using the 16S rRNA sequences. When 16S rRNA sequences of IRP7 and IRP8 strains were compared to related type strains in the NCBI database, similarity levels of 99.65% with *Pseudomonas koreensis* Ps9-14 and 99.44% with *Lysobacter enzymogenes*, respectively, were observed. Moreover, in the constructed maximum likelihood phylogenetic tree (Figure 7), IRP7 and IRP8 were grouped into separate clusters with *P. koreensis* Ps9-14 and *L. enzymogenes*, respectively. Thus, the IRP7 strain was identified as *P. koreensis* and IRP8 was identified as *L. enzymogenes*, and their 16S rRNA sequences were deposited in GenBank with accession numbers MW362840 and MW362396, respectively.

## 4. Discussion

There is an increasing need to establish effective and environmentally friendly control measures for PWD, particularly with limitations on the use of chemical nematicides. Biological control may provide an effective option for management of the disease and has been confirmed to be effective for the management of many other diseases caused by plant-parasitic nematodes [34]. One major mechanism of biological control is induction of resistance by treatment with biological agents [21]. Bacterial treatment has been shown to control infection due to tomato root-knot nematode, *Meloidogyne incognita*, with induction of resistance being the major mode of action [35]. Kim et al. [22], reported isolation and selection of three bacterial strains with the ability to induce systemic resistance in pine callus and seedlings against PWD and confirmed the effect of bacterial treatment on expression of resistance-related genes. In the present study, two bacterial strains, IRP7 and IRP8, isolated from pine forests were selected and used to induce resistance in pine seedlings against PWD. Consistent with the findings of previous studies, the bacterial strains used in this study significantly reduced PWD symptoms in pine seedlings.

Although induction of systemic resistance using biological agents was previously reported against PWD [19,21,22], little is known about the influence of such treatments on the rhizosphere microbiota. The rhizosphere microbiome comprises a very dense microbial community, as the cell and gene count outnumber those of the plant, with up to 10^8^–10^9^ bacteria per gram of soil; rhizosphere symbionts perform critical roles in plants by facilitating nutrient uptake, suppressing colonization by parasitic organisms, modulating plant immunity, and controlling diseases [36,37]. Thus, evaluating the effect of nematode inoculation and resistance-inducing treatments on the structure of the rhizosphere microbiota and analyzing the effect of changes associated with such treatments on the functional microbial taxa could help explain the mechanisms by which biological treatment is useful for managing PWD [23].

The microbial composition and assembly of the rhizosphere microbiota depend on several factors and are largely affected by the interaction with the host plants through the root exudates that act as chemical attractants and repellents, regulating the microbial community [38]. Remarkably, plant roots secrete approximately 5–21% of all photosynthetically fixed carbon in the rhizosphere, comprising a large array of compounds as root exudates [39]. Treatment of plants with active compounds or biological agents might influence the microbial structure in the rhizosphere by causing physiological changes in the plants, such as expression of resistance-related genes, and might also be linked to changes in the root exudates [22,23,40]. Hu et al. [41], reported that the defensive secondary metabolites benzoxazinoids, released by the roots of cereals, are responsible for alterations in the rhizosphere microbiota and consequently several plant physiological changes and signaling leading to herbivore performance inhibition in the next plant generation. In the present study, nematode inoculation, which resulted in a profound effect on pine seedling physiology, as shown by the development of PWD symptoms, had an even more profound effect on the rhizosphere microbial structure, as shown in the beta-diversity measurement, in which nematode-inoculated seedlings were grouped in a cluster separate from the uninoculated groups, regardless of the bacterial treatment.

The rhizosphere of nematode-inoculated seedlings had lower levels of beneficial microbes such as *Paraburkholderia*, *Bradyrhizobium*, *Rhizobacter*, *Lysobacter*, and *Caballeronia*, which are often useful for promoting plant growth and protecting against infections and are known root symbiotics or plant growth promoting rhizobacteria, producing an array of bioactive compounds [42,43,44]. A reduction in the relative abundance of these genera could also contribute to the reduced health status of nematode-inoculated seedlings compared with that of the control. In a previous study, treatment of pine seedlings with resistance-inducing chemical elicitors MeSA and ASM resulted in significant changes in the rhizosphere microbial composition, particularly in ecologically important microbial taxa that were present at low relative abundance [23]. In the present study, treatment with the resistance-inducing bacterial strains did not result in significant shifts in the rhizosphere microbial structure, although it influenced several groups that were present at relatively low abundance.

The rhizosphere of pine seedlings treated with IRP7 after nematode inoculation had higher abundance of *Nitrospirillum*, *Bacillus*, and *Luteibacter* genera, which might be useful for protection against infection, as reported in previous studies. The genome data of the nitrogen fixer and growth promoter *Nitrospirillum*, particularly *N. amazonense*, were previously analyzed and contained several genes coding for cellular properties and metabolic pathways allowing for successful establishment and colonization of plant roots, such as production of siderophores, auxins, polyamines, and autoinducer molecules; biosynthesis of flagellum, σ-, and fimbriae; and development of secretion systems and a complete denitrification system [45]. Members of *the Bacillus* genus have been known to sustain a close association with the rhizoplane and promote plant growth, mainly by facilitating nutrient availability; several strains from this genus have been used as biofertilizers for different crops [46,47,48], and inoculation of *Pinus pinea* with strains belonging to *the Bacillus* genus was reported to enhance plant growth, likely through production of gibberellin and resulting in alteration of the rhizosphere microbial structure [49]. In addition, the genus *Luteibacter* encompasses members that were shown to exhibit plant growth-promotion properties, as indicated in a comparative study with a reference plant growth-promoting strain, in which *Luteibacter* showed superior activity in enhancing plant growth, production of siderophores and 3-indol acetic acid (IAA), and seed germination [50].

In the rhizosphere of pine seedlings treated with IRP8 after nematode inoculation, although the relative abundance of beneficial microbial taxa such as *Paraburkholderia* and *Bradyrhizobium* was lower, a higher relative abundance of the obligate bacterial predators, *Bdellovibrio*, was observed. Members of the *Bdellovibrio* genus are small, highly motile, Gram-negative bacteria that predate other Gram-negative bacteria and have the ability to control plant pathogenic bacteria such as *Pectobacterium* spp. and *Dickeya* spp. causing bacterial soft rot in potato, *Pseudomonas glycinea* causing bacterial blight in soybean, and *Pseudomonas tolaasii* causing brown-blotch lesions on mushrooms [51,52,53]. An increase in the abundance of these predatory microbes could potentially indicate the ability to reduce the pathogenic nematode-carried bacteria that are associated with PWD and that were confirmed to be responsible for symptom development by the production of phytotoxins [6,7]. Although this suggestion needs further confirmation in future studies, it could provide a potential effective management option for PWD.

The selected bacteria were identified as *P. koreensis* IRP7 and *L. enzymogenes* IRP8. Several previous reports have demonstrated the biocontrol activity of *P. koreensis* strains and their ability to antagonize fungal phytopathogens and produce IAA and cytokinin-like compounds [54]. This species is also known for producing biosurfactants that could be used to suppress several plant diseases such as late blight on potato and *Pythium* damping in tomato [55,56]. *Lysobacter enzymogenes* is a chitinolytic species known to possess a wide range of PGPR and biocontrol activities. The antifungal and antinematode activity of these bacteria could be largely attributed to the biosynthesis of chitinase and antibiotics, which degrade the cell wall of chitin-containing organisms including that of nematode eggs [57,58]. Previous studies have reported the biocontrol activity of *L. enzymogenes* against a wide range of plant diseases, including plant parasitic nematodes such as *Caenorhabditis elegans*, *Heterodera schachtii*, *Meloidogyne javanica*, *Pratylenchus penetrans*, and *Aphelenchoides fragariae* [59]. Consistent with our results, induction of resistance by *L. enzymogenes* has also been reported against *Fusarium* head blight and *Bipolaris sorokiniana* leaf spot [60,61].

## 5. Conclusions

The present study reports the biocontrol of PWD using foliar treatment with *P. koreensis* IRP7 and *L. enzymogenes* IRP8 as well as the influence of nematode inoculation and bacterial treatment on the structure of rhizosphere microbiota. Changes occurring in the rhizosphere of pine seedlings upon treatment with the bacterial agents may be linked to PWD control and the improved health status of nematode-inoculated seedlings. To the best of our knowledge, this is the first report on the response of pine rhizosphere microbiota to foliar treatment with resistance-inducing biological agents that result in potential feedback on growth and defense. The present results are considered a step forward toward finding a potential application in the protection against PWD in a sustainable and environmental friendly approach.

## Figures and Tables

**Table 1:** Alpha diversity indices of rhizosphere microbiota from pine seedling samples.

Treatment	OTUs	Chao1	Shannon	Inverse Simpson
C	1145 ± 98.29	1416.73 ± 122.95	7.96 ± 0.62	0.99 ± 0.01
I7	1229 ± 25.91	1438.03 ± 38.25	8.42 ± 0.06	0.99 ± 0.00
I8	1191 ± 159.7	1420.94 ± 177.89	7.79 ± 0.56	0.97 ± 0.02 *
N	1125 ± 57.6	1350.52 ± 82.64	7.82 ± 0.07	0.99 ± 0.00
N+I7	1112 ± 193	1322.57 ± 211.73	7.68 ± 0.73 *	0.98 ± 0.01
N+I8	1081 ± 98.98	1314.23 ± 109.29	7.48 ± 0.21 *	0.97 ± 0.01
Nematode-inoculated	1106 ± 118.81	1329.11 ± 132.70	7.66 ± 0.43	0.98 ± 0.01
Nematode-uninoculated	1183 ± 105.13 *	1425.23 ± 115.09 *	8.06 ± 0.52 *	0.98 ± 0.02

Values are presented as the mean ± standard deviation (*n* = 4). C, negative control; I7, samples treated with *Pseudomonas koreensis* IRP7; I8, samples treated with *Lysobacter enzymogenes* IRP8; N, nematode-inoculated; N+I7, nematode-inoculated samples treated with *P. koreensis* IRP7; N+I8, nematode-inoculated samples treated with *L. enzymogenes* IRP8. * indicates a statistically significant difference at *p* < 0.05, according to Student’s *t*-test between different treatments.

## Data Availability

Bacterial 16S rRNA gene fragments were deposited in NCBI GenBank with accession numbers MW362840 and MW362396. The obtained sequences for the pine rhizosphere microbiota were deposited as a sequence read archive in the National Center for Biotechnology Information database under BioProject ID PRJNA689106.

## Supplementary Materials

The following materials are available online at https://www.mdpi.com/article/10.3390/microorganisms9040688/s1, Table S1: Results of high-throughput sequencing representing the total bases, read count, GC%, Q20%, and Q30%; Figure S1: Relative transcript abundance of the defense-related genes PR-2 family (b-1,3-glucanase), PR-3 family (class IV chitinase), PR-5 family (thaumatin-like protein), PR-9 family (peroxidase), PR-10 family (ribonuclease-like protein), antimicrobial peptides, and metallothionein-like proteins in *Pinus densiflora* calli treated with selected bacterial strains isolated from pine forests. Quantitative real-time polymerase chain reaction (PCR) results from treated calli were normalized relative to untreated calli as a control. The horizontal line represents unchanged gene expression.
