# Supplementary material for: Response of Pine Rhizosphere Microbiota to Foliar Treatment with Resistance-Inducing Bacteria against Pine Wilt Disease
Source: Microorganisms. 2021 Mar 26;9(4):688. doi: 10.3390/microorganisms9040688 (PMC8065550; doi:10.3390/microorganisms9040688)
Supplement: Supplementary file 1 [file microorganisms-09-00688-s001.pdf]

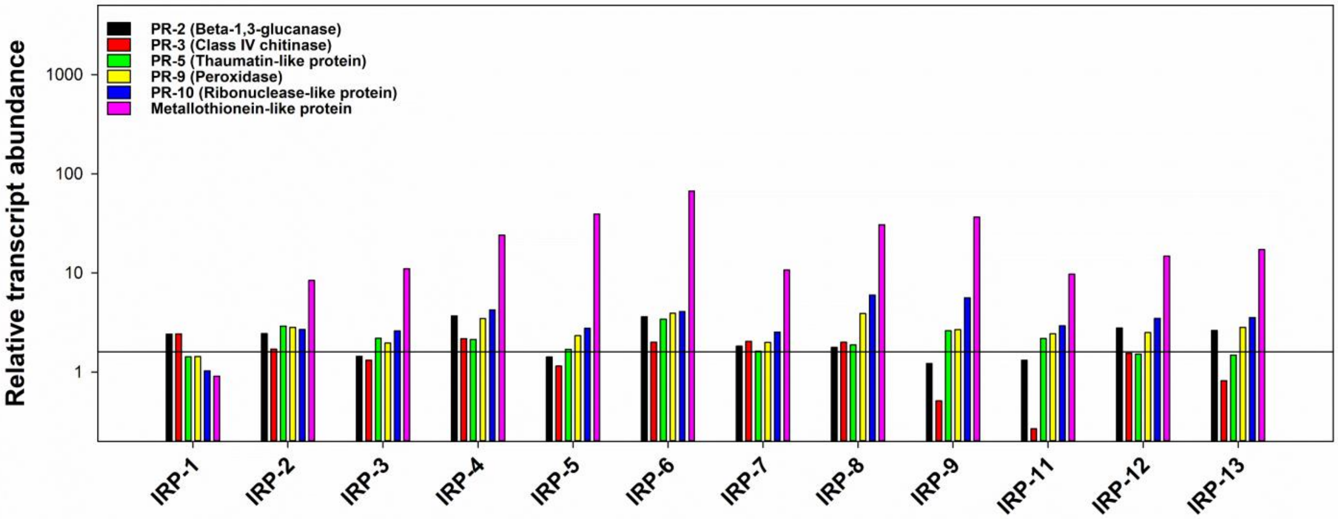

Figure S1. Relative transcript abundance of the defence-related genes PR-2 family (b-1,3-glucanase), PR-3 family (class IV chitinase), PR-5 family (thaumatin-like protein), PR-9 family (peroxidase), PR-10 family (ribonuclease-like protein), antimicrobial peptide and metallothionein-like protein in *Pinus densiflora* calli treated with selected bacterial strains isolated from pine forests. Quantitative real-time PCR results from treated calli were normalized relative to an untreated callus as a control. The horizontal line represents unchanged gene expression.

Table S1. Results of the high throughput sequencing representing the total bases, read count, GC%, Q20% and Q30%

Sample	Total bases	Read count	GC (%)	Q20 (%)	Q30 (%)
C.1	38,381,755	84,870	54.74	98.55	95.23
C.2	42,702,628	94,134	54.41	98.55	95.25
C.3	43,350,263	95,572	54.83	98.58	95.37
C.4	45,499,877	100,657	54.72	98.59	95.34
IRP20.1	43,211,809	95,489	54.69	98.58	95.29
IRP20.2	47,626,696	105,046	54.79	98.6	95.3
IRP20.3	44,501,929	98,381	54.37	98.61	95.37
IRP20.4	43,911,832	97,115	54.62	98.64	95.41
IRP21.1	43,190,112	95,628	54.86	98.52	95.09
IRP21.2	42,920,113	94,802	54.68	98.71	95.62
IRP21.3	42,968,966	94,660	54.8	98.57	95.23
IRP21.4	46,916,335	103,383	54.86	98.7	95.58
NI.1	50,010,132	110,963	54.61	98.72	95.64
NI.2	49,236,449	108,878	54.35	98.61	95.31
NI.3	48,641,503	107,219	54.28	98.46	95.04
NI.4	45,977,229	101,697	54.55	98.63	95.43
N+IRP20.1	45,582,825	100,688	54.06	98.51	95.13
N+IRP20.2	46,636,931	103,055	54.26	98.56	95.17
N+IRP20.3	47,117,369	103,968	54.4	98.54	95.26
N+IRP20.4	51,031,958	112,646	54.38	98.69	95.61
N+IRP21.1	45,448,172	100,449	54.54	98.52	95.12
N+IRP21.2	48,227,030	105,848	54.48	98.54	95.26
N+IRP21.3	49,528,975	109,079	54.37	98.63	95.42
N+IRP21.4	38,022,394	84,016	54.12	98.22	93.97

C, negative control; I20, samples treated with *Pesudomonas koreensis* IRP20; IRP21 samples treated with *Lysobacter enzymogenes* IRP21; N, nematode-inoculated; N+IRP20, nematode-inoculated samples treated with *P. koreensis* IRP20; N+IRP21, nematode-inoculated samples treated with *L. enzymogenes* IRP21.
